# The Future of Serum Therapeutics

**Published:** 1894-11-24

**Authors:** 


### THE FUTURE OF SERUM THERAPEUTICS.

Recent developments in therapeutics are beginning to make people ask in all seriousness where it is all to end. We have long been accustomed to vaccination, and while this remained an isolated fact, difficult as it might be to understand how it could possibly operate, at any rate it was a small matter, was got over in infancy, and was soon forgotten. People, however, look on the matter from rather a different standpoint when they find themselves threatened with inoculation with animal fluids for the treatment of a whole series of diseases. Hydrophobia, tuberculosis, tetanus, cholera, diphtheria, small-pox, are among the infective diseases for the prophylaxis or treatment of which inoculations have recently been practised; while among other maladies for which animal juices have been injected may be mentioned myxoedema, cretinism, diabetes, various forms of debility, and a whole crowd of skin diseases. No excuse, then, is needed for the anxiety which is felt as to the future of this line of treatment. Average man has considerable confidence in his stomach as a selective agent. He knows well enough that he, in common with many other animals, constantly puts into that long-suffering organ many things which must somewhat astonish its mucous membrane, but he sees and, if he does not understand, he knows by experience that, what with stomach, and liver, and glands, the body does somehow manage to manufacture a standard blood, notwithstanding the rubbish on which it is nourished, and man, therefore, not without reason, trusts greatly to his stomach. Serum therapeutics, however, are independent of the limitations imposed by a man's digestive capacity. The drug, whatever it may be, goes straight into the blood.

All the locks and bars imposed by nature to keep the blood in its normal state are evaded by the doctor with a syringe; and the whole digestive tract, with its dependent glands, stands on guard in vain when antitoxine serum slips in by the back door, viâ the subcutaneous tissues. In truth this is a serious matter, and imposes on the doctor a far heavier responsibility than ever attended those more simple measures which, after all, were finally subjected to the arbitration of the stomach. It must be confessed that in regard to serum therapeutics a large superstructure is being raised upon a comparatively small basis, for large as are the number of observations, and positive as may be the evidence that the serum of certain animals under the influence of certain poisons is protective against the products brewed by certain organisms, we are sadly in the dark as to the method by which this neutralisation or immunisation, as it is called, is produced. Pathology is in this matter considerably ahead of therapeutics, and in endeavouring to estimate in what direction future developments should be hoped for, we may be excused bringing to bear some dash of that particular form of the scientific imagination which goes by the name of common sense. We do not wish —no one wishes—to be tied down to what is likely; but if we are to grope at all from the point at which we stand, the question, What is likely? must enter into our calculations. Now, all analogy suggests that the pathologists are right in saying that each microbe brews, maybe as a waste product, a poison of its own. All our vegetable drugs are but illustrations of the fact that each form of life tends to grow a product of its own, and we can well believe that each pathogenic organism produces a something which by its action on the body causes the symptoms of its own disease. The other side of the question, however, is not so simple; it is by no means likely that one body should be able to brew all kinds of different chemical products so as to be able to neutralise the various toxins produced by the infinite range of pathogenic organisms to whose attacks man is exposed. Man's body would indeed be a sort of magic bottle if, at the word of command, it could produce all these various compounds. The products which result from cell-activity are very numerous, possibly even more numerous than chemists who analyse them are willing to admit, for, after all, what the chemist finds is rather the stuff that living tissue turns into when dead than what it actually is while carrying on the processes of life; but, numerous as they may be, there is no proof that all sorts of different chemical antidotes can be produced by the body to neutralise the various toxic substances elaborated by microbic life. It seems impossible then to admit that finality will have been reached when an anti-toxin has been discovered for each possible toxin, for, so far as probability goes, it seems much more likely, if such a phrase is admissible, that in the production of all these anti-toxins there must be some common factor, some one substance on which the virtue of all depends, and the probabilities are very great that this one substance is but an excess of some protective material normal to health, rather than something fresh formed in response to the irritation of so many different pathogenic toxins.

Much has been said against the doctrine of phagocytosis, but even those who object most strongly to the suggestion that phagocytes can enclose and digest microbes admit that leucocytes secrete substances having bactericidal power. These clearly are not forced, but natural, products, and are the means by which, in ordinary health, the incursions of microbes are prevented; and it may well be that it is by an interaction between the varied toxins and these standard products of leucocytes that the anti-toxins are produced, for whereas toxins can be brewed in test-tubes, anti-toxins require the living blood. If then beyond and behind the anti-toxins there be some one substance engaged in the production of them all, we may, perhaps, look forward to the time when we shall see order in place of the present chaos. Much in the same way as one alkali will neutralise all acids, so we may hope to find that some one substance or some mode of vital action will neutralise or destroy all toxins. The knowledge that these toxins are definite chemical compounds makes us vastly discontented with the current methods of counteracting them by means of vague anti-toxins, resulting from we know not what action in the organism, and if it is ever right to use the imagination in scientific matters, we may fairly look forward to the time when the protective substance, whatever it may be, will be shown to be something normal to the body, and when the treatment of infective disease will be undertaken, not by the injection of products of the malady, but by an increase of the natural resistance of the healthy body.

